# Analysis of the efficacy of endovascular treatment with foam sclerotherapy for pelvic congestion syndrome with ultrasound assessment

**DOI:** 10.1590/1677-5449.202301782

**Published:** 2024-10-21

**Authors:** Natália Miranda Gava, Amanda Santana Silva, Gustavo Sasso Benso Maciel, Márcia Porto Assis, Carlos André Daher Santos

**Affiliations:** 1 Hospital Santa Casa de Misericórdia de Vitória, Vitória, ES, Brasil.; 2 Universidade Federal do Espírito Santo – UFES, Vitória, ES, Brasil.; 3 Hospital Universitário Cassiano Antônio de Morais – HUCAM Vitória, ES, Brasil.

**Keywords:** varicose veins, pelvic pain, therapeutic embolization, sclerotherapy

## Abstract

**Background:**

Pelvic Congestion Syndrome is an important cause of pelvic pain in adult women, leading to reduced quality of life, absenteeism from work, anxiety, depression, and sexual disorders.

**Objectives:**

To evaluate the response to endovascular treatment for pelvic varicose veins using foam sclerotherapy and outline the profile of patients with this diagnosis followed up at the Hospital das Clínicas Cassiano Antônio Moraes, Universidade Federal do Espírito Santo, Brazil.

**Methods:**

Based on review of medical records, this retrospective descriptive study analyzes the profile and response of patients undergoing endovascular treatment for pelvic varicose veins by foam sclerotherapy. The variables analyzed include age, weight, height, body mass index, parity, pelvic pain complaints, ultrasound criteria comparing the diameter of pelvic vessels before and after the procedure, and presence of venous reflux on transvaginal Doppler ultrasound.

**Results:**

The sample of patients analyzed had an average age of 43.3 years old, a mean of 2.95 gestations, and a mean BMI of 25.37kg/m^2^. Ultrasound assessment after the intervention indicated a statistically significant reduction (*p*-value < 0.005) in the caliber of the parauterine vessels, with mean diameters of 6.34 mm on the right and 7.26 mm on the left before the procedure and 4.37 mm and 4.56 mm respectively afterwards.

**Conclusions:**

Foam sclerotherapy reduced the caliber of pelvic varicose veins in the study sample. The results were similar to those of other endovascular treatment methods for this comorbidity. Further prospective studies to assess the response to this intervention are necessary to establish it as an effective option for treatment of pelvic congestion syndrome.

## INTRODUCTION

Pelvic congestion syndrome (PCS) is an important cause of chronic pelvic pain in the female population. It manifests clinically as a variety of symptoms. The most common are dyspareunia, pain/weight in the lower abdomen, vulvar edema, and rectal discomfort.^1,2^ Classically, these symptoms tend to worsen after long periods standing and in response to physical activity or sexual intercourse.^3^

Pelvic varicose veins are a frequent finding in adult women, affecting approximately 10% of the general female population, 60% of whom are symptomatic.^1^ The prevalence of PCS is greater among women with a history of at least one prior labor,^2^ suggesting correlations between development of the disease and both hormonal factors related to pregnancy and extrinsic venous compression by the gravid uterus.^4^

Diagnosis is suspected in the presence of a suggestive clinical history and/or observation of vulvar varicose veins.^2^ However, diagnosis is confirmed with a supplementary radiological study, whether by transvaginal ultrasonography (USG) with Doppler, computed tomography or magnetic resonance imaging of the pelvis, or laparoscopy or phlebography of gonadal vessels.^1,3^

Some studies suggest hormonal pharmacological treatment is a therapeutic option, but this strategy has not been tested in well-designed clinical trials with significant numbers of patients.^4^ Surgical treatment options that have been described are venous ligation via laparoscopy, hysterectomy, and oophorectomy,^3,4^ but endovascular approaches, which are minimally invasive interventions, stand out from other options because of their safety and results.

It has thus been proposed that the treatment of choice for PCS is endovascular, consisting of access to the pelvic veins via phlebography, followed by injection of an embolizing agent, such as platinum coils and vascular plugs, with the objective of provoking permanent occlusion and thrombosis of the embolized area.^2-6^

There are a number of studies in the literature showing the efficacy of treatment with coils, such as those published by Siqueira et al.^3^ , Hocquelet et al.^5^ and Guilora et al.^6^ , but there is still a lack of objective data evaluating endovascular interventions with administration of other sclerosing agents, such as interventions using foam only, for example.

Therefore, the objective of this study is to assess the efficacy of endovascular treatment of PCS with foam sclerotherapy using ultrasonographic examination before and after intervention.

## METHODS

A longitudinal, retrospective analytical study was designed to assess the efficacy of endovascular treatment with foam sclerotherapy for PCS.

The endovascular interventions analyzed were performed at a tertiary University Hospital with its own catheterization laboratory and all cases were female patients treated on Brazil’s Unified Health System (SUS - Sistema Único de Saúde).

Permission to collect data from patients’ medical records was granted by the Research Ethics Committee at the Universidade Federal do Espírito Santo, Hospital Universitário Cassiano Antônio de Moraes, which analyzed the project and approved it under consolidated opinion nº 3.237.958 and Ethics Appraisal Submission Certificate 03820718.3.0000.5071.

The study analyzed data from all patients who underwent elective endovascular treatment with foam sclerotherapy from August 2015 to March 2018, with an initial sample size of n = 30.

It is important to point out that the procedure was only proposed for patients who met the following ultrasonographic criteria: parauterine vessels with caliber > 5 mm and venous reflux on Doppler, or with caliber > 6 mm regardless of the presence of reflux.

These patients’ medical records were reviewed to evaluate the profile of the study population, recording the following demographic data: age, weight, height, body mass index (BMI), parity, and complaints of pelvic pain (including reports of dyspareunia and/or dysmenorrhea).

**Figure 1:**
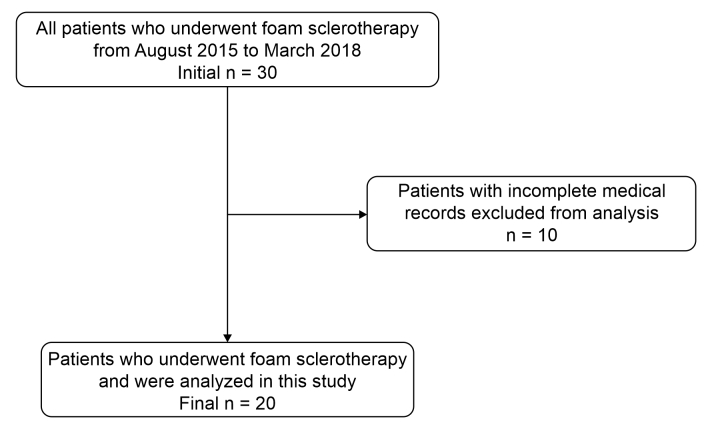
Flow diagram illustrating the process of selecting the convenience sample analyzed.

After the initial review of the medical records, patients were excluded from the study if their records were considered incomplete. i.e., cases in which all of the information listed above were not correctly described on the medical record, as were cases in which the patient was lost to follow-up, resulting in a final sample of 20 patients. Figure 1 contains the patient selection flow diagram.

It is relevant to mention that the sample of patients was selected for assessment exclusively from among those who underwent foam sclerotherapy interventions for treatment of pelvic varicose veins in the catheterization laboratory at the Hospital das Clínicas Cassiano Antônio Moraes, Universidade Federal do Espírito Santo, during the period selected for analysis, thus constituting a convenience sample.

For the purposes of comparison, considering a standard deviation (SD) of 5.9161 and a standard error of 1.3228, calculated from the available sample described above (n = 20), the ideal minimum sample size for this study would be 76.84, i.e., approximately 77 participants. As such, the sample actually studied is the equivalent of about 1/4 of this value, which should be taken into consideration, since a small sample increases the possibility that the results observed would be affected by biases.

The efficacy of interventions was assessed by analyzing the diameter of the pelvic vessels before and after the procedure (using the paired *t* test) and presence of venous reflux on preoperative and postoperative transvaginal Doppler. Additionality, in order to standardize the analysis, the mean time elapsed between endovascular intervention and postoperative ultrasound assessments was also calculated.

Patients’ clinical responses were assessed on the basis of a comparison of percentage presence of pelvic pain preintervention and postintervention, using the McNemar test.

The variables analyzed related to the procedure were number of interventions, type of sclerosing agent used (monoethanolamine or polidocanol), volume of sclerosing agent administered, and route of access used.

All of the procedures analyzed in this study were performed in the cardiovascular intervention, diagnosis, and treatment center at the University Hospital, with aseptic technique and according to the following protocol: under local anesthesia (lidocaine 2%), a venous puncture is performed in the right cubital fossa, with insertion of a 6 Fr/11 cm introducer. Next, a hydrophilic guidewire 0.035” is inserted and a 5 Fr PM catheter is used for infusion of contrast (Optray 300) for phlebography and catheterization of the gonadal veins. Next, foam produced with 2 ml of sclerosing agent (monoethanolamine oleate 5% or polidocanol 3%) and 8 ml of air is administered via injection into the gonadal vessel, using the Tessari technique.^7^ Finally, control phlebography of the gonadal and iliac vessels is performed, completing the procedure.

The statistical analysis was conducted by an independent statistician and results were considered significant when p < 0.05 with a 95% confidence interval (CI). Categorical variables were expressed as absolute and relative frequencies and distributions of metric variables were assessed after calculating measures of central tendency and variability (mean and standard deviation).

## RESULTS

The sample of patients included in the study comprised women aged 27 to 68 years, with a mean age of 43.4 (SD 9.12), as described in Table 1. The sample included one patient who had never given birth and one who had had 10 gestations, with mean parity of 2.95 (SD 2.31) for the whole sample. Mean patient height was 1.60 m (SD 0.07) and mean weight was 64.83 kg (SD 13.96), with mean BMI of 25.37 kg/m^2^ (SD 5.55).

**Table 1:** Characteristics of the sample: age, parity, and anthropometric data.

Variables	Mean	Standard deviation	95% CI
Age	43.40	9.12	39.41-43.39
Gestations	2.95	2.31	1.94-3.96
Weight (kg)	64.83	13.86	58.76-70.90
Height (m)	1.60	0.07	1.57-1.63
BMI	25.37	5.55	22.94-27.80

**Subtitle:** BMI = body mass index; CI = Confidence Interval

The mean diameter of gonadal vessels before the procedure was 6.34 mm (SD 1.96) on the right and 7.26 mm (SD 1.87) on the left, while the equivalent values after intervention were 4.37 mm (SD 1.48) and 4.56 mm (SD 1.44) respectively, with a p value of 0.000, as shown in Table 2.

**Table 2:** Diameter of pelvic varicose veins before and after foam sclerotherapy, with analysis by the paired *t* test.

**Variables**	**Time**	**Mean**	**Standard deviation**	**p**
Diameter, right	Before	6.34	1.96	0.000024
	After	4.37	1.48	
Diameter, left	Before	7.26	1.87	0.000008
	After	4.56	1.44	

Before the procedure, 80% (n = 16) of the patients had bilateral gonadal vein reflux and 20% (n = 4) had reflux on the left only, as shown in Table 3. After the procedure, bilateral reflux was found in 20% (n = 4) of cases, reflux on the left only was observed in 30% (n = 6) and reflux on the right only was observed in 15% (n = 3), while no reflux was observed in the Doppler study in the remaining 35% (n=7) of patients.

**Table 3:** Venous reflux in pelvic varicose veins before and after foam sclerotherapy.

**Variables**	**Preprocedure**		**Postprocedure**	
	**n**	**%**	**n**	**%**
Reflux				
Bilateral	16	80.0	4	20.0
Left	4	20.0	6	30.0
Right	-	-	3	15.0
None	-	-	7	35.0
**Total**	**20**	**100.0**	**20**	**100.0**

The mean time elapsed between endovascular treatment and the control Doppler examination was 6.2 months (SD 3.45).

Table 4 shows the results of the comparison of pelvic pain complaints at different times during follow-up. Ninety-five percent of the patients (n = 19) reported pain before the procedure, whereas only 30% (n = 6) still complained of pain after the intervention, with p < 0.05.

**Table 4:** Complaints of pelvic pain before and after foam sclerotherapy, with analysis by the McNemar test.

**Pelvic pain**	**Preprocedure**		**Postprocedure**		**p**
	**n**	**%**	**n**	**%**	
Yes	19	95.0	6	30.0	0.000244
No	1	5.0	14	70.0	
**Total**	**20**	**100.0**	**20**	**100.0**	**-**

Regarding data related to the procedures, it was found that 70% (n = 14) of the sample underwent a single intervention, while the other 30% (n = 6) underwent a second treatment session. The sclerosing agent used was monoethanolamine oleate in 70% (n = 14), polidocanol was used in another 25% (n = 5), and both substances were used in 5% (n = 1). The volume of sclerosing agent employed in the interventions ranged from 10 to 25 ml, with a mean of 18.2 ml (SD 4.11). In 85% (n = 17) of the patients, sclerosis was achieved by catheterization of the gonadal veins bilaterally, and in 15% (n = 3) only the left vein was catheterized. The access route used most often was the right cephalic vein (n = 6), followed by the right brachial vein (n = 4) and the right intermediate vein (n = 4). Variables related to the procedure are shown in Tables 5 and 6.

**Table 5:** Procedural variables: number of interventions, type of sclerosing agent, side of intervention and puncture site.

**Variables**	**n**	**%**
Second treatment		
No	14	70.0
Yes	6	30.0
Type of sclerosing agent		
Monoethanolamine	14	70.0
Polidocanol	5	25.0
Monoethanolamine/polidocanol	1	5.0
Side		
Left	3	15.0
Bilateral	17	85.0
Puncture site		
Right cephalic	6	30.0
Right brachial	4	20.0
Right intermediate	4	20.0
Right basilic	3	15.0
Right arm	2	10.0
Right cephalic/right intermediate	1	5.0
**Total**	**20**	**100.0**

**Table 6:** Volume of sclerosing agent used in interventions.

**Variable**	**Mean**	**Standard deviation**	**95% CI**
Volume (ml)	18.20	4.11	16.40-20

**Subtitle:** CI = Confidence Interval

With regard to direct complications of the procedure, a single case of thrombophlebitis at the puncture site was found in the medical records analyzed.

## DISCUSSION

Pelvic congestion syndrome is an important cause of chronic pelvic pain in the young, adult, economically active female population that causes reduced quality of life, work absenteeism, anxiety, depression, and sexual disorders.^3,5^

The mean age of the patients in the study sample was 43.4 years, with a mean of 2.95 gestations. In a meta-analysis published in 2016 that combined the results of 22 studies (a total of 1,308 patients) of endovascular treatment for pelvic varicose veins, the mean age of patients ranged from 32 to 51 years, while mean parity ranged from 0.9 to 3.5 gestations.^8^

With regard to the pathophysiology of this disease, despite attempts to establish a correlation between the female hormonal component, transitory compression of pelvic vessels by the gravid uterus, and development of the syndrome,^4^ few studies attempting to identify the cause of pelvic congestion syndrome have been conducted to date. Moreover, the findings of studies that have been conducted were not replicated in other studies corroborating them. It is known, however, that PCS can be related to specific anatomic abnormalities, such as compression of the left renal vein by the superior mesenteric artery, known as Nutcracker syndrome, or by compression of the left common iliac vein by the right common iliac artery, known as May-Thurner Syndrome.^1^ Involvement of the venous system is more frequently observed on the left because of the right angle at which the ovarian vein enters the left renal vein, promoting venous reflux.^3^

The radiological arsenal employed in the context of this disease is used both to identify venous insufficiency and to rule out other chronic pelvic pain differential diagnoses, such as endometriosis, adenomyosis, adherences, and inflammatory diseases. The diagnostic criteria proposed in the literature include gonadal vein diameter exceeding 8 mm, parauterine varicose veins with diameter of at least 5 mm, and/or presence of venous reflux on Doppler, dynamic magnetic resonance angiography, or phlebography.^5^

Some studies suggest hormonal pharmacological treatment, with dihydroergotamine, medroxyprogesterone, etonogestrel implant, flavonoids, or goserelin, as possible therapeutic options, but this strategy still lacks supporting evidence from well-designed clinical trials with significant numbers of patients.^4^ Additionally, while hormonal treatment is noninvasive, it does not achieve good long-term results,^3,5^ according to what is demonstrated by the scientific data available to date.

Venous ligation via laparoscopy, hysterectomy, and oophorectomy have also been described as treatment options,^3,4^ but the safety and the results obtained with endovascular treatments, which are minimally invasive interventions, have meant that these methods stand out from the rest.^4^

The first report of endovascular treatment of pelvic varicose veins using coils was published in 1993 by Edward et al.,^9^ and since then this method has been increasingly accepted as the first-choice treatment for the condition, both because of its efficacy and because of the low rate of complications and rapid return to daily activities.^1-3,5^

A study published in 2003 assessed 106 women with PCS, divided into three groups: 52 underwent embolization with coils, 32 underwent hysterectomy with unilateral oophorectomy, and 34 underwent hysterectomy with bilateral oophorectomy and hormone replacement. Endovascular treatment proved significantly more effective for controlling pelvic pain than the other methods and also had a lower rate of complications.^10^

At our service, we employed a different approach to the standard protocol described in the literature, which proposes embolization using metallic coils. In our interventions we used monoethanolamine or polidocanol as sclerosing agents, with a mean volume of 18.2 ml (SD 4.11) injected after catheterization of the gonadal vein.

Ultrasonographic assessment after endovascular intervention using this protocol showed a statistically significant reduction (p < 0.05) in the caliber of the parauterine vessels. The mean diameter observed was 6.34 mm (SD 1.96) on the right and 7.26 mm (SD 1.87) on the left before the procedure and 4.37 mm (SD 1.48) and 4.56 mm (SD 1.44) respectively after treatment. In a study by Siqueira et al.,^3^ the mean caliber of vessels before coil embolization was 6.7 mm (SD 1.6), reducing to 4.9 mm (SD 1.5) after the procedure.

Another study, published in 2003, assessed the ultrasonographic response to treatment of pelvic varicose veins with percutaneous injection of 3% sodium tetradecyl sulfate, without coils. In that study, the mean caliber of vessels before the procedure was 4.5 mm on the right and 6.5 mm on the left and after the procedure calibers of 3.19 mm and 4.5 mm were observed, with statistical relevance.^11^

In addition to ultrasonographic assessment, in terms of the clinical response of patients, a categorical assessment of pain symptoms, using the McNemar test, demonstrated a reduction from 70 to 30% in complaints of pelvic pain after foam sclerotherapy (p < 0.05), enabling us to conclude there was a trend for clinical success of the treatment.

During the period analyzed, from August 2015 to March 2018, 30% of the sample (6 out of 20 patients) needed a second treatment session, which is similar to the number in the study by Siqueira et al.,^3^ in which four (18.18%) patients needed a repeat intervention.

The literature reports that the incidence of complications from coil embolization is rare, with the most frequent (3% of cases) type related to puncture site,^8^ such as hematoma and phlebitis,^2^ and considers migrations to be the most severe, reported in around 1.6% of cases.^3^ These data are corroborated by the observations in our sample, in which the only complication reported was a single case of thrombophlebitis at the puncture site.

Despite the statistically significant results found in this study, which are compatible with the findings of other, more robust studies, it should be pointed out that a small number of patients underwent foam sclerotherapy during the period analyzed, which was reduced further still by loss of cases because of incomplete follow-up or inadequate recording of data on medical records, resulting in a small final sample, which restricts the possibilities for extrapolation of the results described here to the general population. Moreover, the retrospective nature of the study confers disadvantages inherent to the format, such as information bias and less control over the variables analyzed.

## CONCLUSIONS

Analysis of the efficacy of foam sclerotherapy for treatment of pelvic congestion syndrome with comparison of preoperative and postoperative assessments by ultrasonography revealed that this method of endovascular intervention effectively reduced the caliber of pelvic varicose veins and occurrence of venous reflux in these vessels. It was also possible to confirm a significant reduction in complaints of pelvic pain among the patients after the procedure.

However, while the results in the sample analyzed were statistically significant, it should be emphasized that external validation of the findings is impossible because of the small size of the study.
