# Supplementary material for: Analysis of the efficacy of endovascular treatment with foam sclerotherapy for pelvic congestion syndrome with ultrasound assessment
Source: J Vasc Bras. 2024 Oct 21;23:e20230178. doi: 10.1590/1677-5449.202301782 (PMC11529999; doi:10.1590/1677-5449.202301782)
Supplement: Translation

# Translation: Análise da eficácia do tratamento endovascular com escleroterapia por espuma na síndrome de congestão pélvica através de avaliação por ultrassonografia

**DOI:** 10.1590/1677-5449.202301781

**Authors:** Natália Miranda Gava, Amanda Santana Silva, Gustavo Sasso Benso Maciel, Márcia Porto Assis, Carlos André Daher Santos

**Affiliations:** 1 Hospital Santa Casa de Misericórdia de Vitória, Vitória, ES, Brasil.; 2 Universidade Federal do Espírito Santo – UFES, Vitória, ES, Brasil.; 3 Hospital Universitário Cassiano Antônio de Morais – HUCAM Vitória, ES, Brasil.

**Keywords:** varizes, dor pélvica, embolização terapêutica, escleroterapia

## Abstract

**Contexto:**

A síndrome de congestão pélvica (SCP) é uma importante causa de dor pélvica em mulheres adultas, levando à redução da qualidade de vida, abstenção no trabalho, ansiedade, depressão e distúrbios sexuais.

**Objetivos:**

Avaliar a resposta ao tratamento endovascular por escleroterapia com espuma para varizes pélvicas e traçar o perfil das pacientes com esse diagnóstico acompanhadas no Hospital das Clínicas Cassiano Antônio Moraes, da Universidade Federal do Espírito Santo.

**Métodos:**

Com base na revisão de prontuários, este estudo descritivo retrospectivo analisa o perfil e a resposta das pacientes submetidas ao tratamento endovascular de varizes pélvicas por escleroterapia com espuma. As variáveis analisadas incluem idade, peso, altura, índice de massa corporal (IMC), paridade, queixa de dor pélvica e critérios ultrassonográficos, comparando o diâmetro dos vasos pélvicos pré e pós-procedimento, e a presença de refluxo venoso ao Doppler transvaginal.

**Resultados:**

As pacientes analisadas tinham em média 43,3 anos, 2,95 gestações e IMC de 25,37kg/m^2^. A avaliação ultrassonográfica após intervenção indicou uma redução estatisticamente significativa (valor de p < 0,005) do calibre dos vasos parauterinos, com diâmetro médio de 6,34 mm à direita e 7,26 mm à esquerda antes do procedimento e, respectivamente, 4,37 mm e 4,56 mm após o procedimento.

**Conclusões:**

A escleroterapia com espuma reduziu o calibre das varizes pélvicas na amostra estudada, com resultados semelhantes aos de outras modalidades de tratamento endovascular dessa comorbidade. A realização de mais estudos prospectivos para avaliação da resposta a essa intervenção se faz necessária para firmá-la como uma opção eficaz para o tratamento da SCP.

## INTRODUÇÃO

A síndrome de congestão pélvica (SCP) é uma importante causa de dor pélvica crônica na população feminina, que se manifesta clinicamente por uma variedade de sintomas. Entre os mais comuns, estão: dispareunia, dor/peso em abdome inferior, edema vulvar e desconforto retal^1,2^ . Classicamente, esses sintomas tendem a piorar após longos períodos de pé, com atividade física ou intercurso sexual^3^ .

Varizes pélvicas são achados frequentes em mulheres adultas, acometendo aproximadamente 10% da população feminina geral, das quais 60% são sintomáticas^1^ . A prevalência da SCP é maior entre mulheres com história de ao menos um trabalho de parto prévio^2^ , sugerindo uma correlação tanto entre os fatores hormonais relacionados à prenhez quanto com a compressão venosa extrínseca pelo útero gravídico^4^ com o desenvolvimento da doença.

O diagnóstico é suspeitado a partir de uma história clínica sugestiva e/ou pela observação de varizes vulvares^2^ . A confirmação diagnóstica, entretanto, é obtida através de um estudo radiológico complementar, seja por ultrassonografia (USG) transvaginal com Doppler, tomografia computadorizada ou ressonância nuclear magnética da pelve, laparoscopia ou flebografia de vasos gonadais^1,3^ .

Alguns estudos sugerem o tratamento farmacológico hormonal como uma das possibilidades terapêuticas, porém tal estratégia ainda carece de comprovação por ensaios clínicos bem desenhados e com número significativo de pacientes^4^ . Ligaduras venosas por via laparoscópica, histerectomia e ooforectomia também são opções de tratamento cirúrgico descritas^3,4^ , porém a segurança e os resultados das abordagens endovasculares, que são intervenções minimamente invasivas, fizeram com que esses tratamentos se destacassem quando comparados aos demais.

O tratamento de escolha para a SCP, portanto, tem sido proposto por via endovascular, consistindo no acesso às veias pélvicas por flebografia, seguida por injeção de material embolizante, como molas de platina e plugues vasculares, a fim de causar oclusão permanente e trombose do território embolizado^2-6^ .

A literatura traz alguns estudos que mostram a eficácia do tratamento com molas, como nos de Siqueira et al.^3^ , Hocquelet et al.^5^ e Guilora et al.^6^ , porém ainda há uma carência de dados objetivos na avaliação de intervenções endovasculares com aplicação de outros agentes esclerosantes, por exemplo com o uso isolado de espuma.

Portanto, este estudo objetiva avaliar a eficácia do tratamento endovascular com escleroterapia por espuma na SCP através da avaliação ultrassonográfica pré e pós-intervenção.

## MÉTODOS

Estudo analítico longitudinal e retrospectivo, desenhado com o objetivo de avaliar a eficácia do tratamento endovascular por escleroterapia com espuma na SCP.

As intervenções endovasculares em questão foram realizadas em hospital universitário de nível terciário, com serviço de hemodinâmica próprio, e englobaram apenas pacientes do sexo feminino vinculadas ao Sistema Único de Saúde (SUS).

A coleta de informações nos prontuários foi realizada com a aprovação do Conselho de Ética em Pesquisa da Universidade Federal do Espírito Santo – Hospital Universitário Cassiano Antônio de Moraes que, após apreciação do projeto, aprovou-o no parecer consubstanciado nº 3.237.958, Certificado de Apresentação de Apreciação Ética 03820718.3.0000.5071.

Foram avaliadas todas as pacientes submetidas ao tratamento endovascular por escleroterapia com espuma de maneira eletiva, no período entre agosto de 2015 e março de 2018, somando um n inicial de 30 pacientes.

É importante destacar que o procedimento foi proposto apenas para as pacientes que atendessem aos seguintes critérios ultrassonográficos: vasos parauterinos com calibre > 5 mm e refluxo venoso ao Doppler, ou com calibre > 6 mm independente da presença de refluxo.

Os prontuários médicos dessas pacientes foram revisados para avaliação do perfil da população estudada, observando-se os seguintes dados demográficos da amostra: idade, peso, altura, índice de massa corporal (IMC), paridade e queixa de dor pélvica (incluindo relatos de dispareunia e/ou dismenorreia).

Após revisão inicial dos prontuários, foram excluídas do estudo as pacientes cujos registros foram considerados incompletos. Ou seja, nos casos em que não fossem apreciáveis todas as informações citadas acima adequadamente descritas em prontuário e/ou nos casos em que houve perda de seguimento, resultando num n final de 20 pacientes. O processo de determinação da amostra estudada se encontra ilustrado na Figura 1.

**Figura 1:** Fluxograma demonstrando o processo de seleção da amostra de conveniência analisada.

É pertinente ressaltar que a seleção das pacientes avaliadas resultou exclusivamente do levantamento do número de intervenções de escleroterapia por espuma para tratamento de varizes pélvicas realizadas pelo serviço de hemodinâmica do Hospital das Clínicas Cassiano Antônio Moraes da Universidade Federal do Espírito Santo no período pretendido para a análise, constituindo assim uma amostra de conveniência.

Para fim de comparação, considerando um desvio-padrão (DP) de 5,9161 e um erro padrão de 1,3228, calculados a partir da amostra disponível descrita (n = 20), o tamanho mínimo ideal de amostra para este estudo equivale a 76,84, ou seja, aproximadamente 77 participantes. Assim sendo, a amostra estudada corresponde a quase 1/4 desse valor, devendo isso ser levado em consideração, pois uma amostra reduzida aumenta a chance de que os resultados observados sejam afetados por vieses.

Para avaliação da eficácia das intervenções foram analisados o diâmetro dos vasos pélvicos antes e após o procedimento (pelo teste de t-pareado) e a presença de refluxo venoso ao Doppler transvaginal pré e pós-operatórios. Além disso, para padronização da análise, foi calculado o tempo médio entre a realização das intervenções endovasculares e das avaliações pós-operatórias por ultrassonografia.

A resposta clínica das pacientes foi avaliada baseada na comparação percentual relativa à presença de dor pélvica pré e pós-intervenção, realizada pelo teste de McNemar.

Dentre as variáveis referentes ao procedimento, foram avaliados: número de intervenções, tipo de esclerosante utilizado (monoetanolamina ou polidocanol), volume de esclerosante aplicado e via de acesso.

Todos os procedimentos incluídos neste estudo foram realizados no setor de intervenção, diagnóstico e tratamento invasivo em doenças cardiovasculares do hospital universitário, sob técnica asséptica e obedecendo ao seguinte protocolo: sob anestesia local (lidocaína 2%), é realizada punção venosa em fossa cubital direita, com instalação de introdutor 6 Fr/11 cm. Em sequência, é realizada passagem de fio guia hidrofílico 0,035” e cateter MP de 5 Fr com infusão de contraste (Optray 300) para flebografia e cateterismo das veias gonadais. A seguir, é aplicada injeção de espuma formada por 2 ml de agente esclerosante (oleato de monoetanolamina 5% ou polidocanol 3%) e 8 ml de ar em vaso gonadal, sob técnica de Tessari^7^ . Finalmente, é realizada a flebografia de controle dos vasos gonadais e ilíacos ao final do procedimento.

A análise estatística foi efetuada por um estatístico independente e, para determinação de relevância estática nos resultados avaliados, o valor de p foi considerado significativo quando < 0,05, com um intervalo de confiança (IC) de 95%. As variáveis categóricas foram expressas pelas suas frequências absolutas e relativas, enquanto a distribuição das variáveis métricas foi avaliada pela determinação de suas medidas de posição central e variabilidade (média e DP).

## RESULTADOS

A amostra de pacientes incluídas no estudo, caracterizada na Tabela 1, foi composta por mulheres entre 27 e 68 anos, com uma idade média de 43,4 (DP 9,12); Entre essas, havia uma paciente nulípara e uma paciente com história de 10 gestações, sendo a paridade média da amostra de 2,95 (DP 2,31). A altura média das pacientes foi aferida em 1,60 m (DP 0,07) e o peso médio de 64,83 kg (DP 13,96), de forma que o IMC médio calculado foi de 25,37 kg/m2 (DP 5,55).

**Tabela 1:** Caracterização da amostra: idade, paridade e antropometria.

**Variáveis**	**Média**	**Desvio-padrão**	**IC 95%**
Idade	43,40	9,12	39,41-43,39
Gestações	2,95	2,31	1,94-3,96
Peso (kg)	64,83	13,86	58,76-70,90
Altura (m)	1,60	0,07	1,57-1,63
IMC	25,37	5,55	22,94-27,80

**Legenda**: IMC = índice de massa corporal; IC = Intervalo de Confiança

O diâmetro médio dos vasos gonadais antes do procedimento foi de 6,34 mm (DP 1,96) à direita e 7,26 mm (DP 1,87) à esquerda, enquanto os valores registrados após a intervenção foram de, respectivamente, 4,37 mm (DP 1,48) e 4,56 mm (DP 1,44), com um valor de p de 0,000, estando esses achados exibidos na Tabela 2.

**Tabela 2:** Diâmetro das varizes pélvicas pré e pós-escleroterapia por espuma, conforme avaliação pelo teste t-pareado.

**Variáveis**	**Momentos**	**Média**	**Desvio-padrão**	**Valor de p**
Diâmetro D	Pré	6,34	1,96	0,000024
	Pós	4,37	1,48	
Diâmetro E	Pré	7,26	1,87	0,000008
	Pós	4,56	1,44	

Antes do procedimento, conforme demonstrado na Tabela 3, 80% (n = 16) das pacientes apresentavam refluxo venoso gonadal bilateral e 20% (n = 4) apenas à esquerda. Após o procedimento, registrou-se refluxo bilateral em 20% (n = 4) dos casos, apenas à esquerda em 30% (n = 6) e apenas à direita em 15% (n = 3), enquanto nos 35% (n=7) restantes não foi observado refluxo ao estudo com Doppler.

**Tabela 3:** Refluxo venoso nas varizes pélvicas pré e pós-escleroterapia por espuma.

**Variáveis**	**Pré-procedimento**		**Pós-procedimento**	
	**n**	**%**	**n**	**%**
Refluxo				
Bilateral	16	80,0	4	20,0
Esquerda	4	20,0	6	30,0
Direita	-	-	3	15,0
Não	-	-	7	35,0
**Total**	**20**	**100,0**	**20**	**100,0**

O tempo médio calculado entre a execução do tratamento endovascular e a realização do Doppler de controle foi de 6,2 meses (DP 3,45).

Delineada na Tabela 4, encontra-se a comparação entre as queixas de dor pélvica em diferentes momentos do acompanhamento. Entre as pacientes, 95% (n = 19) relataram dor antes do procedimento, enquanto após a intervenção a queixa se manteve presente em apenas 30% (n = 6), com um valor de p < 0,05.

**Tabela 4:** Queixa de dor pélvica pré e pós-escleroterapia por espuma, conforme avaliação pelo teste de McNemar.

**Dor Pélvica**	**Pré-procedimento**		**Pós-procedimento**		**Valor de p**
	**n**	**%**	**n**	**%**	
Sim	19	95,0	6	30,0	0,000244
Não	1	5,0	14	70,0	
**Total**	**20**	**100,0**	**20**	**100,0**	**-**

No que tange aos dados referentes ao procedimento, constatou-se que 70% (n = 14) da amostra foi submetida a uma única intervenção, enquanto os outros 30% (n = 6) foram submetidos a uma segunda sessão do tratamento. Em 70% (n = 14) dos casos, o agente esclerosante utilizado foi o oleato de monoetanolamina, em outros 25% (n = 5) se utilizou o polidocanol e em 5% (n = 1) foram aplicadas as duas substâncias. O volume de esclerosante utilizado nas intervenções variou de 10 a 25 ml, com uma média de 18,2 ml (DP 4,11). Em 85% (n = 17) das pacientes a esclerose foi realizada por cateterismo das veias gonadais bilateralmente, e em 15% (n = 3) apenas da veia esquerda. A via de acesso mais utilizada foi a veia cefálica direita (n = 6), seguida das veias braquial direita (n = 4) e intermédia direita (n = 4). As variáveis envolvidas no procedimento estão demonstradas nas Tabelas 5 e 6.

**Tabela 5:** Variáveis do procedimento: número de intervenções, tipo de esclerosante, lateralidade da abordagem e sítio de punção.

**Variáveis**	**n**	**%**
Segundo tratamento		
Não	14	70,0
Sim	6	30,0
Tipo de esclerosante		
Monoetanolamina	14	70,0
Polidocanol	5	25,0
Monoetanolamina/polidocanol	1	5,0
Lado		
Esquerda	3	15,0
Bilateral	17	85,0
Sítio de punção		
Cefálica D	6	30,0
Braquial D	4	20,0
Intermédia D	4	20,0
Basílica D	3	15,0
Braço Direito	2	10,0
Cefálica D/intermédia D	1	5,0
**Total**	**20**	**100,0**

**Tabela 6:** Volume de esclerosante utilizado nas intervenções.

**Variáveis**	**Média**	**Desvio-padrão**	**IC 95%**
Volume (ml)	18,20	4,11	16,40-20

**Legenda:** IC = Intervalo de Confiança

No que diz respeito a complicações diretas do procedimento, entre os prontuários avaliados, foi registrado um único caso de tromboflebite no local de punção.

## DISCUSSÃO

A SCP é uma importante causa de dor pélvica crônica na população feminina adulta, jovem e economicamente ativa, que leva à redução da qualidade de vida, abstenção no trabalho, ansiedade, depressão e distúrbios sexuais^3,5^ .

A idade média das pacientes da amostra estudada foi de 43,4 anos, com paridade média de 2,95 gestações. Em uma metanálise publicada em 2016, que reuniu os resultados de 22 estudos (1.308 pacientes, no total) para tratamento endovascular de varizes pélvicas, a idade média das pacientes esteve entre 32 e 51 anos, enquanto a paridade média variou de 0,9 a 3,5 gestações^8^ .

Quanto à fisiopatologia da doença, apesar das tentativas de estabelecer uma correlação entre o componente hormonal feminino, a compressão transitória dos vasos pélvicos pelo útero gravídico e o desenvolvimento da síndrome^4^ , até hoje, poucas pesquisas foram desenvolvidas visando identificar a causa da síndrome de congestão pélvica. Para além disso, os achados desses estudos não foram replicados em outros trabalhos que os corroborem. É sabido, entretanto, que a SCP pode se relacionar a anormalidades anatômicas específicas, como a compressão da veia renal esquerda pela artéria mesentérica superior, conhecida como síndrome Nutcracker, ou pela compressão da veia ilíaca comum esquerda pela artéria ilíaca comum direita, nomeada síndrome de May-Thurner^1^ . O acometimento do sistema venoso à esquerda é mais frequentemente observado devido ao ângulo reto em que a veia ovariana se insere na veia renal esquerda, favorecendo o refluxo venoso^3^ .

O arsenal radiológico aplicado no contexto da doença se presta tanto à identificação da insuficiência venosa quanto à exclusão de diagnósticos diferenciais de dor pélvica crônica, como endometriose, adenomiose, aderências e doenças inflamatórias. Os critérios diagnósticos propostos pela literatura incluem: diâmetro maior que 8 mm da veia gonadal, varizes parauterinas com diâmetro de pelo menos 5 mm e/ou com presença de refluxo venoso ao Doppler, angiografia dinâmica por ressonância ou flebografia^5^ .

Alguns estudos sugerem o tratamento farmacológico hormonal, com di-hidroergotamina, medroxiprogesterona, implante de etonogestrel, flavonóides ou goserelina, como uma das possibilidades terapêuticas, porém tal estratégia ainda carece de comprovação por ensaios clínicos bem desenhados e com número significativo de pacientes^4^ . Além disso, apesar de não invasivo, o tratamento hormonal não apresenta bons resultados a longo prazo^3,5^ , de acordo com o demonstrado pela produção científica disponível até o momento.

Ligaduras venosas por laparoscopia, histerectomia e ooforectomia também são opções terapêuticas descritas^3,4^ , porém a segurança e os resultados dos tratamentos endovasculares, que são intervenções minimamente invasivas, fizeram com que essa modalidade se destacasse quando comparada às demais^4^ .

O primeiro relato de tratamento endovascular de varizes pélvicas com molas data de 1993, publicado por Edward et al.^9^ , e desde então vem sendo cada vez mais aceita como primeira escolha no tratamento desta afecção, tanto por sua efetividade quanto pela baixa taxa de complicações e rápido retorno das pacientes às atividades diárias^1-3,5^ .

Um estudo publicado em 2003 avaliou 106 mulheres com SCP, dividindo-as em três grupos: 52 submetidas a embolização com molas, 32 submetidas a histerectomia com ooforectomia unilateral, e 34 submetidas a histerectomia com ooforectomia bilateral e reposição hormonal. O tratamento endovascular mostrou-se significativamente mais eficaz no controle da dor pélvica em relação aos demais, além de apresentar uma taxa menor de complicações^10^ .

Em nosso serviço, adotamos uma abordagem diferente do protocolo padrão encontrado na literatura, que propõe embolização com molas metálicas. Na nossa abordagem, utilizamos a monoetanolamina ou polidocanol como agentes esclerosantes, com volume médio de 18,2 ml (DP 4,11) injetados após cateterismo da veia gonadal.

A avaliação ultrassonográfica após a intervenção endovascular aplicando esse protocolo traz uma redução estatisticamente significativa (valor de p < 0,05) do calibre dos vasos parauterinos. O diâmetro médio encontrado foi de 6,34 mm (DP 1,96) à direita e 7,26 mm (DP 1,87) à esquerda antes do procedimento e, respectivamente, 4,37 mm (DP 1,48) e 4,56 mm (DP 1,44) após o tratamento. Já no estudo de Siqueira et al.^3^ , o calibre médio dos vasos antes da embolização com mola foi de 6,7 mm (DP 1,6) e de 4,9 mm (DP 1,5) após o procedimento.

Outro estudo, publicado em 2003, avaliou a resposta ultrassonográfica ao tratamento das varizes pélvicas com injeção percutânea de tetradecilsulfato de sódio a 3%, sem uso de molas. Nele, o calibre médio dos vasos antes do procedimento era de 4,5 mm à direita e 6,5 mm à esquerda, observando-se, após o procedimento, calibres de 3,19 mm e 4,5 mm, respectivamente, com relevância estatística^11^ .

Além da avaliação ultrassonográfica, no que diz respeito à resposta clínica das pacientes, uma avaliação categórica dos sintomas álgicos, utilizando o teste de McNemar, demonstrou uma redução de 70 para 30% referente à queixa de dor pélvica antes e após a escleroterapia por espuma (valor de p < 0,05), nos permitindo inferir uma tendência ao sucesso clínico do tratamento.

Durante o período avaliado, entre agosto de 2015 a março de 2018, 30% da amostra (6 das 20 pacientes) necessitou de uma segunda abordagem durante o tratamento, número semelhante ao encontrado no estudo de Siqueira et al.^3^ , em que quatro (18,18%) pacientes necessitaram de nova abordagem.

A literatura traz como rara a incidência de complicações decorrentes da embolização com molas, sendo as mais frequentes (3% dos casos) aquelas relacionadas ao sítio de punção^8^ , como hematomas e flebites^2^ , e considerando como as mais temidas as migrações, relatadas em cerca de 1,6% dos casos^3^ . Esses dados corroboram os observados em nossa amostra, na qual a única complicação relatada foi a de um caso de tromboflebite no sítio de punção.

Apesar dos resultados estatisticamente significativos encontrados neste estudo, que são condizentes com os achados de outras pesquisas com maior robustez, é necessário pontuar que o n restrito de pacientes submetidas à escleroterapia por espuma no período analisado, reduzido ainda pela perda de casos devido a seguimento incompleto ou registro inadequado de informações em prontuário, resultou numa amostra final pequena, o que restringe a possibilidade de extrapolação dos resultados aqui descritos para a população geral. Além disso, o caráter retrospectivo do estudo denota desvantagens inerentes à sua formatação, como o viés de informação e um menor controle sobre as variáveis avaliadas.

## CONCLUSÃO

Ao estudar a eficácia da escleroterapia por espuma no tratamento da síndrome de congestão pélvica, avaliações comparativas pré e pós-operatórias por ultrassonografia verificaram que essa modalidade de intervenção endovascular reduziu efetivamente o calibre das varizes pélvicas e a ocorrência de refluxo venoso nesses vasos. Além disso, foi possível verificar uma redução significativa da queixa de dor pélvica referida pelas pacientes antes e após o procedimento.

Entretanto, apesar de na amostra avaliada os resultados obtidos serem estatisticamente significativos, é preciso ressaltar a impossibilidade de validação externa de tais achados considerando a dimensão restrita deste estudo.
